# The battle for autophagy between host and influenza A virus

**DOI:** 10.1080/21505594.2021.2014680

**Published:** 2021-12-30

**Authors:** Ao Zhou, Wenhua Zhang, Xia Dong, Mengyun Liu, Hongbo Chen, Bin Tang

**Affiliations:** a Hubei Provincial Center of Technology Innovation for Domestic Animal Breeding, College of Animal Science and Nutritional Engineering, Wuhan Polytechnic University, Wuhan, 430023, P.R. China; b Department of Chemistry, School of Basic Medical College, Southwest Medical University, Luzhou, 646100, People’s Republic of China

**Keywords:** Autophagy, antiviral response, host, influenza virus proteins, drug

## Abstract

Influenza A virus (IAV) is an infectious pathogen, threatening the population and public safety with its epidemics. Therefore, it is essential to better understand influenza virus biology to develop efficient strategies against its pathogenicity. Autophagy is an important cellular process to maintain cellular homeostasis by cleaning up the hazardous substrates in lysosome. Accumulating research has also suggested that autophagy is a critical mechanism in host defense responses against IAV infection by degrading viral particles and activating innate or acquired immunity to induce viral clearance. However, IAV has conversely hijacked autophagy to strengthen virus infection by blocking autophagy maturation and further interfering host antiviral signalling to promote viral replication. Therefore, how the battle for autophagy between host and IAV is carried out need to be known. In this review, we describe the role of autophagy in host defence and IAV survival, and summarize the role of influenza proteins in subverting the autophagic process as well as then concentrate on how host utilize antiviral function of autophagy to prevent IAV infection.

## Introduction

Influenza A virus (IAV) is an important infectious pathogen causing significant morbidity and mortality to result in enormous economic losses in the world [1,2]. The genome of IAV possesses eight segmented molecules encoding for multiple proteins[3], including viral polymerase proteins (PB1, PB2 and PA) and nucleoprotein (NP), two surface glycoproteins (HA and NA), two matrix proteins (M1 and M2) and two non-structural proteins (NS1 and NS2) [4,5], suggesting that IAV can constantly acquire genetic mutation or re-assortment to evade host immune response and increase the resistance to antiviral drugs [6,7]. In addition, the transmission of IAV between swine, avian and human also resulted in potential pandemic with airborne transmissible viruses [8,9]. Recently, a new influenza virus (G4 viruses) isolated from pig was an re-assortant Eurasian avian-like (EA) H1N1 virus that contains the segment of 2009 pandemic (pdm/09) virus[10] and replicated in human airway epithelial cells[10]. Additionally, like other viruses, IAV relied on host cellular functions to support its replication and hijack the host cell machinery to complete its life cycle. Therefore, in order to develop more efficacious antiviral countermeasures against IAV, the game of “hide and seek” between influenza A virus and host needed to be understood.

**Figure 1.**
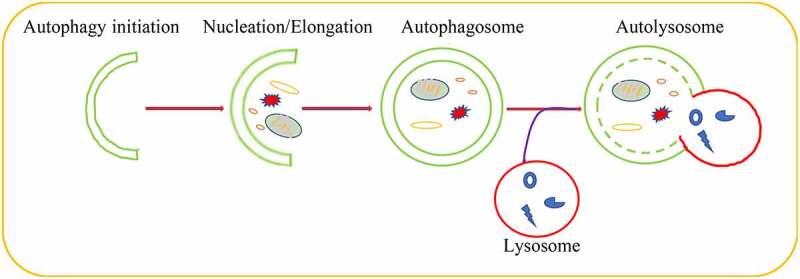
Schematic overview of the autophagic pathway.

Autophagy played a significant role in maintaining cellular homeostasis[11]. As a conserved critical degradation process, autophagy targeted aberrant cellular cytoplasmic constituents and damaged organelles, proteins aggregate as well as the invading pathogens for lysosomal degradation [12,13]. Autophagy induction started from phagophore formation regulated by autophagy-related (ATG) proteins that recruited to membranes of many organelles to initiate autophagy[14]. Subsequently, the damaged organelles, misfolded proteins and protein aggregates were encircled by phagophore to lead to the nucleation and the formation of pre-autophagosomal structures (PASs), whose elongation formed autophagosomes through recruiting other membrane structure [15–18]. As a consequence, autophagosomes fused with lysosomes to form autolysosome that degraded the captured contents by lysosomal proteases[19] (Figure 1). Growing evidences indicated that each step of autophagic pathway was regulated by a large number of molecules or pathways [20–22].

As a fundamental cellular process, autophagy was important for host and virus. Host utilizes autophagy to clear the intracellular waste and invading pathogens for maintaining organismal homeostasis[23]. Autophagy activation inhibited pseudorabies virus infection[24] and overexpressing of autophagy-related genes reduced viral replication and Sindbis virus-induced mortality [25,26]. Additionally, autophagy was also an important regulator in innate immunity and the control of inflammasomes [27–29]. However, viruses escaped autophagia degradation or induce autophagy for virus survival and spread with the evolved specific mechanisms [30,31]. Hepatitis C virus (HCV) replication, maturation and release depended on autophagy machinery[32]. Moreover, HCV-induced autophagy impaired innate immune response for its spread[33]. In the early life cycle of virus, Foot-and-mouth disease virus (FMDV) and Japanese encephalitis virus promoted autophagy initiation to help virus trafficking and viral infection [34–37].Taken together, the mechanism on the battle for autophagy between virus and host was very sophisticated. Therefore, in this review, we focused on dual character of autophagy in response to influenza virus refection and discuss the value of autophagy-related antiviral compounds against IAV.

## Influenza A virus utilizes autophagic machinery to enhance viral infection

Numerous studies deepened our understanding regarding the autophagy machinery during virus infection over the past years, but whether influenza virus utilized autophagy for their benefit was still unclear. In 2009, the first report was shown that IAV infection induced the formation autophagosomes and increased the conversion of LC3I to LC3II, which is an autophagosome marker protein, illustrating that autophagy was involved in IAV infection[26]. Subsequently, large amounts of evidences showed that autophagy was indispensable to IAV infection whatever lower or highly pathogenic avian influenza strains [27–30] (Table 1).

**Table 1.** Different strains of influenza virus regulate autophagy formation in different cell types

Viral Strains	Infected Cells	Multiplicity of infection (MOI)	Reference
A/WSN/33 (H1N1)	MDCK; A549	0.05	36
A/duck/Hubei/Hangmei01/2006(H5N1)	A549	0.1	37
A/swine/HeBei/012/2008 (H9N2)	A549	2	38
A/swine/HeBei/012/2008 (H9N2)	A549	1	39
A/Jilin/9/2004 (H5N1)	MEF	10	40
A/Quail/Hong Kong/G1/97 (H9N2/G1) A/Hong Kong/54/98 (H1N1) A/HongKong/415,742/09 (S-OIV)	Primary blood macrophages	2	41
A/Hong Kong/8/68 (H3N2)	Memory B cells	5	42
A/WSN/33(H1N1)	MDCK	5	43
A/PR/8/34(H1N1)	hiPSCs	5	44
A/mallard/Huadong/S/2005 (H5N1) A/Ck/SH/F/98(H9N2) A/PR8/34(H1N1)	CEF; DF1	0.1	45
A/Aichi/68 (H3N2)	A549; MLE-12; MDAMC; HaCat	0.6 or 0.8	48
A/PR/8/34(H1N1)	A549	1	52
A/WSN/33(H1N1)	A549	0.01	53
A/PR/8/34 (H1N1)	Hela	1	54
A/new.Coledonia/20/1999(H1N1) A/Jilin/9/2004(H5N1)	A549	4	68
A/Zhejiang/2/2009 (H1N1)	BMDCs	4	74
A/Hong Kong/2108/2003 (H9N2) A/Nanjing/108/2009 (H1N1)	A549; Hela; HPMECs; HUVECs	1	92
A3/Beijing/30/95(H3N2)	A549; Ana-1	100TCID_50_	124

MDCK: Madin-Darby canine kidney; A549: human lung epithelial cells; CEF: primary chick embryo fibroblasts; DF1: chicken fibroblast cells; MLE-12: mouse lung epithelial cells; MDAMC: human breast carcinoma cells; HaCat: human keratinocyte cells; hiPSCs: human-induced pluripotent stem cells; Ana-1: murine macrophage; BMDCs: bone‐marrow‐derived dendritic cells; MEF: mouse embryonic fibroblasts; Hela: human cervical carcinoma cell

It has been well-documented that the fusion of autophagosome with lysosome is essential for eliminating superfluous and harmful components as well as maintaining cellular metabolism [38,39]. But influenza virus (H1N1, H3N2) inhibited autophagy maturation to interfere with autophagic antiviral activity through inducing the accumulation of autophagosome and blocking the formation of autolysosome[40]. It has been known that IAV infection caused significantly cell death to exacerbate inflammation and respiratory failure for supporting efficient viral replication and propagation at the late of IAV infection[41]. Interestingly, IAV-induced autophagosome accumulation induced led to enhance IAV-induced apoptosis[42]. Moreover, IAV could also induce autophagic cell death through suppressing the expression of mTOR, which is the key negative regulator of autophagy[43]. IAV-induced apoptosis as a mechanism for virus spread in late infection needed autophagy activation[44], indicating that IAV strength autophagy-induced apoptosis to promote viral replication. In addition, IAV infection could reduce the phosphorylation levels of AKT, TSC2 and mTOR to trigger autophagy initiation, then IAV-induced autophagy degraded the protease sensitive antioxidant SOD1 to result in oxidative stress, which is important for IAV pathogenicity [45–52]. Autophagy-deficient cell model used in viral infection showed that IAV regulated autophagy to increase viral genomic RNAs by decreasing the phosphorylation of mTOR, 4E-BP1 and S6 at the infection early stage and promoting the phosphorylation of p70S6K at the infection late[53].

**Figure 2.**
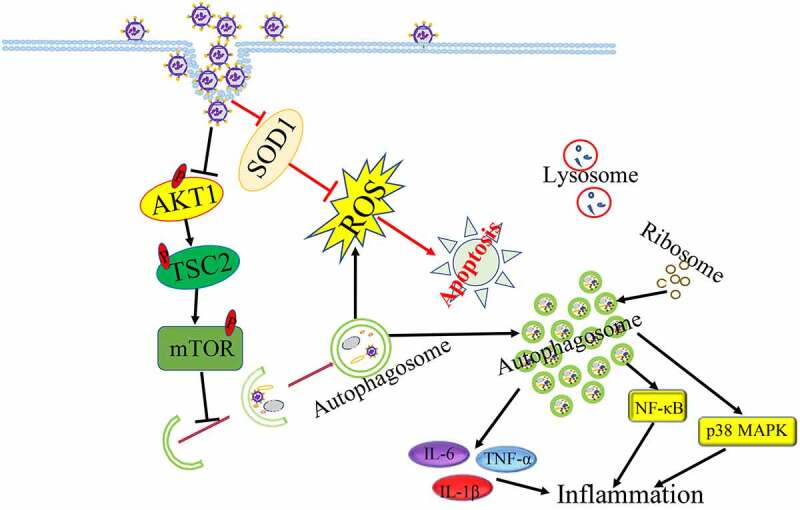
Influenza virus induced autophagy to promote its replication.

Additionally, IAV-induced autophagy increased inflammatory and immune components degradation to promote virus infection. Induction of autophagy under IAV infection led to excessive inflammation to exacerbate acute lung injury induced by IAV through increasing the expression of proinflammatory cytokines or activating NF-κB and p38 MAPK signalling pathways [54,55]. In addition, IAV-induced autophagy limited interferon-β (IFN-β) singling and reduced ISG expression that benefited IAV infection[56]. Moreover, IAV can also affect the composition of the autophagosomal proteome and resulted in the mis-localization of ribosomal proteins, viral proteins and viral mRNA in autophagosomes, suggesting that autophagosomes might be the place of viral protein biosynthesis[57]. Taken together, in order to survival and infection, Influenza virus could utilize different autophagy machineries to regulate host cellular response characteristics in different infection phrases (Figure 2).

## The roles of viral proteins in IAV-regulated autophagy

It has been clear that IAV utilizes autophagy machinery for virus infection. Thus, better understanding how the viral proteins are involved in IAV-regulated autophagy will be critical.

Growing evidences have shown that influenza virus encodes a number of proteins, including ten classical influenza proteins (PB1, PB2, NP, HA, PA, NA, M1, M2, NS1, and NS2) and other proteins via frame-shifts and complementary sequences[58]. IAV matrix protein 2 (M2) was the first reported viral protein to regulate autophagy. The M2 proteins as an ion channel protein formed an acid-activated proton channel to promote viral endocytosis and the vRNP released into host cell nucleus for the synthesis of mRNA, and M2 also promoted the recruitment of the internal viral proteins and vRNA for virus assembly and budding by interacting with M1 protein at late infection [59,60]. The expression of M2 protein prevented autophagy maturation by interfering the fusion of autophagosomes with lysosomes and leading to the accumulation of autophagosomes[40]. Co-immunoprecipitation assay found the interaction between M2 protein and Beclin1, which was an autophagy protein and was responsible for autophagosome formation [61,62]. In addition, IAV infection resulted in the re-localization of LC3 to the plasma membranes and M2 protein was found to colocalize with LC3 at the same time. Further evidence was uncovered that the cytoplasmic tail of M2 protein contain a conserved LC3-interacting region (LIR), which pull LC3 protein to the plasma membrane, suggesting that the interaction of M2 and LC3 block autophagic flux and lead to the autophagosome close to the plasma membrane for the generation of virus particles [60,63]. Interestingly, the M2 protein was capable of increasing Ca^2+^ concentration and ROS production to support autophagosome formation, which in turn increased MAVS aggregations and enhanced MAVS-mediated innate immunity to result in exaggerated inflammation or cytokine storm[64]. Taken together, M2 was crucially important to autophagy initiation and immaturity induced by IAV infection, and M2-induced autophagy in turn blocked the elimination of excessive ROS and increased the excessive immune response to benefit viral infection.

Influenza virus non-structural protein 1(NS1) was also reported to participate in autophagy initiation. The NS1 protein was known to inhibit apoptosis early in infection for virus replication[65]. Subsequently, IAV lacking NS1 poorly stimulated the autophagic process, indicating that the NS1 protein is involved in IAV-regulated autophagy. But NS1 could not induce autophagy alone and need to utilize the synthesis of HA and M2 to initiate the formation of autophagosome[66]. However, there was controversial about the interaction of NS1 with autophagy, another report showed that IAV infection with NS1 mutant (R38AK41A and Y89F) could observe LC3 accumulation, indicating that NS1 might block autophagosomes formation through using the dsRNA-binding activity of NS1 and activating PI3K-Akt singnaling[67]. NS1 inhibited the co-localization LC3 with Rab11a to prevent the sequestration of vRNA complexes in autophagosome for virus spread[67]. We think that different roles of NS1 on autophagy results from the different phase of IAV infection. At early infection time, NS1 may assist M2 with autophagy initiation, while NS1 blocks the engulfment of viral RNP complexes to increase viral assembly and buds, but future studies need to be investigated.

Influenza virus hemagglutinin (HA) protein bound surface receptors to initiate the infectious cycle and could also stimulate the increase of LC3-II as well as regulate the induction of autophagic cell death in infected cells [66,68]. Moreover, The HA protein bound with HSP90AA1 to inhibit the AKT/mTOR signalling pathway and inducing complete autophagy to degrade innate immunity factors in early infection[69]. In addition, HSP90AA1 was also involved in Influenza virus nucleoprotein (NP) protein-induced autophagy, which overexpression of the NP proteins alone leads to the increase of LC3-II and a redistribution of LC3 from the cytosol to autophagosomes vesicles[70]. Due to the interaction of HSP90AA1 with PB2, a subunit of IAV polymerase complex, the expression of NP protein increased the expression of HSP90AA1 and the binding of HSP90AA1 to PB2 to support influenza vRNA synthesis at early time points of infection. The NP protein significantly downregulated the phosphorylation levels of AKT, mTOR, FKHR, and pS706 K to support autophagy initiation[39].

**Figure 3.**
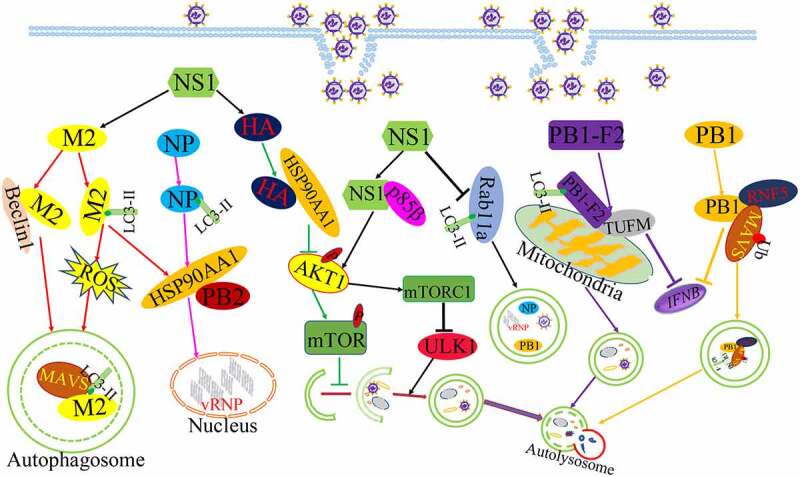
The working model of the roles of different viral proteins in the regulating of autophagy with IAV.

Other influenza virus proteins have been also known to participate in autophagy. IAV polymerase complex subunit PB1 can mediated RNF5 and NBR1-depandent autophagic degradation of MAVS to disrupt MAVS -medicated innate signalling pathway at the early infection[71]. Upon IAV infection, PB1-F2 protein translocated into the mitochondria inner membrane space by interacting with TUFM to accumulation, which accelerates the mitochondrial fragmentation and triggers mitophagy, and PB1-F2-induced mitophagy conversely stimulated MAVS degradation to suppress the type I IFN production and to impair cellular innate immunity [72,73]. Based on the data above, we found that different viral proteins of IAV exhibited a variety of functions in IAV-regulated autophagy. IAV utilized the speciality of different viral proteins to regulate autophagy in difference stages of IAV life cycle (Figure 3).

## Host regulates autophagic machinery against influenza virus infection

Autophagy is the key process in host antiviral response. Host autophagy delivers cellular damaged substrates and virial particles for lysosomal degradation; on the other hand, autophagy integrates with cellular antiviral signaling to block viral replication and spread.

**Figure 4.**
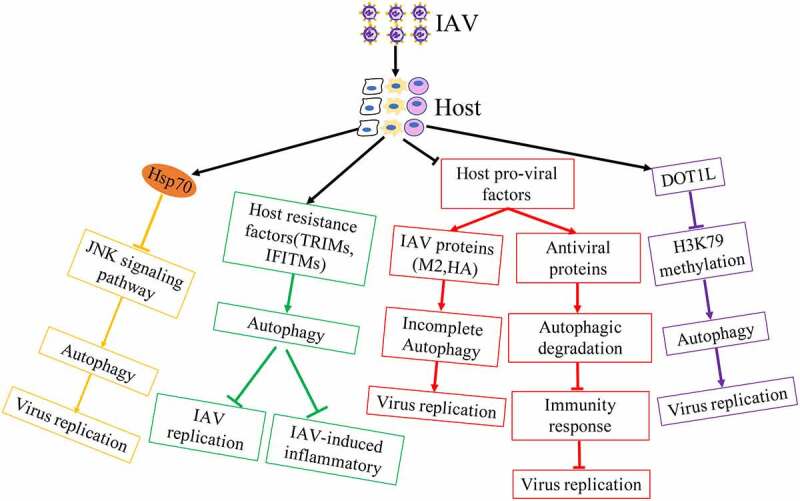
Host regulates autophagy against influenza virus infection.

Although influenza virus has evolved multiple strategies to utilize autophagy for viral infection, host is also able to regulate autophagy machinery against IAV infectious (Figure 4).

Autophagy increased the localization of H1N1 virus in lysosomes to activate TLR signaling and then induces Th cell differentiation in bone-marrow-derived DCs (BMDCs) against IAV [74,75]. In addition, autophagy promoted the accumulation of vRNP aggregates located in autolysosomes for degradation in mammalian cells with influenza virus containing avian PB2^75^.

Although IAV induced complete autophagy to degrade antiviral factors for viral replication by IAV PB1-F2 protein[72], host IL-36γ limited autophagy to upregulate antiviral immunity for blocking IAV replication in the early stages of IAV infection[76]. Heat shock protein 70 inhibited the activity of IAV ribonucleoprotein, the interaction between IAV M1 protein and vRNP and blocked the replication of IAV [77,78]. Additionally, Hsp70 overexpression resulted in low levels of activated JNK, which inhibited IAV-induced autophagy and virus replication [79,80], suggesting that host has the ability to utilize cellular mechanism to regulate autophagy for interfering the synthetic of viral proteins. In addition, host antiviral factors played important roles against IAV replication. Host restriction factor TUFM inhibited the replication of IAV and interacted with influenza viral PB2 protein in mitochondrial by promoting completely autophagy[81].

It has been reported that TRIM proteins are critical in managing several antiviral signalling pathways, including autophagy related pathways against influenza virus[82]. TRIM23 utilized its GTPase activity and E3 ligase activity to activate TBK1 and increased the phosphorylation of p62 for the degradation of virus particles[83]. TRIM28 had been proved to promote autophagy through SUMOylation of PIK3C3 with the assembly of the PIK3C3-BECN1 complex[84]. TRIM28 was phosphorylated and interacted with CTIF to inhibit viral replication by blocking aggresome formation, which promoted the capsids uncoating of influenza virus [85,86], suggesting that TRIM28 blocked viral uncoating thought inhibiting ubiquitin-dependent aggresome formation, and then induce misfolded protein and the viral protein into autolysosome for degradation at the early viral cycle. Receptor interacting protein kinase2 (RIPK2) restricted IAV-induced exacerbated production of IL-18 that reducing IL-18 expressing can ameliorate lung damage and promote survival, thereby dampening inflammasome activation through mitophagy in IAV-infected cells and mice[87]. Interferon-induced transmembrane proteins (IFITMs) acted as restriction factors mediating cellular resistance to IAV replication. Additionally, IFITM3 had the higher expression in resident memory CD8^+^T cells whose survival was dependent on autophagy to suppress IAV infection. IFITM3 also promoted the autophagic degradation of IRF3 to block IAV-induced immoderate immune responses [88–90]. Activation of sphingosine 1-phosphate receptor 1 (S1PR1), the receptor of an immunomodulatory small molecule S1P, blocked IAV-induced cytokine storm and down-regulated the number of virus-specific T cells. Overexpression of S1PR1 decreased p65 phosphorylation and translocation into the nucleus, leading to the inactivation of NF-κB signalling, subsequently suppressing IAV-induced autophagy and inflammatory [91–93]. It has been known that IAV infection lead to hypoxia and HIF-1α played a key role in hypoxia. The increase of HIF-1α level could interfere with AMPKα-ULK1 signaling-dependent autophagy to conversely block viral replication[94]. Host factors CDN1163, an activator of SERCA that regulated transport of calcium ions, reduced autophagosome accumulation caused by IAV to repair autophagic flux. Activation of CDN1163 and SERCA interfered inflammatory cytokines and chemokines production during IAV infection by regulating MAPK-JNK pathway[95].

Epigenetic modifications participated in IAV-medicated autophagy. DOT1L, an inhibitor of the specific H3K79 methylase, inhibited influenza virus replication by regulating the target proteins (Rubicon, TRIM25 and Bcl-3) methylated by H3K79 [96,97]. Down-regulation of Rubicon inhibited IAV replication by increasing types I IFN secretion or autophagy maturation induction, because previous research showed that Rubicon depletion induced autophagosome maturation and the degradation of autophagy substrate p62 [98,99]. TRIM25 mediated Lys 63-linked ubiquitination of RIG-I and strengthened the production of type I interferon to suppression of IAV replication[100]. Bcl-3, a negative regulator of NF-κB activation, enhanced the autophagy process[101]. Galectins as soluble pattern recognition receptors played key roles in pathogen recognition and the regulation of immune homeostasis. It was all known that the glycosylation of HA and NA of influenza virus was critical for viral infection. Galectin-1 was proved to reduce viral replication by directly binding to the envelope glycoproteins of IAV and inhibiting viral hemagglutination activity and infectivity[102]. Moreover, Galectin-1 could also weaken IAV-induced acute lung injury [103,104]. Additionally, previous reports showed that Galectins was capable of trigger complete autophagy by inhibiting the mTOR signalling and its downstream autophagy machinery [105–107]. It was shown that NA and M2 of IAV blocked autolysosome formation via the de-glycosylation of lysosome-associated membrane proteins (LAMPs) for lysosome rupture or the accumulation of autophagosome [108,109]. Thus, we speculated that Galectin might weaken IAV-induced lysosome damage to increase the formation of autolysosome for suppressing IAV infection, although further mechanism needs to be uncovered.

## Anti-influenza virus compounds inhibit influenza virus infection via autophagic machinery

It was well-known that autophagy machinery and autophagy-related proteins were involved in IAV infection. Down-regulation of autophagy-related proteins, including Beclin1, ATG3, ATG5, ATG7 and LC3 significantly suppressed IAV infection, indicating that pharmacological approaches targeting autophagy process and autophagy-related proteins will have a huge potential to inhibit IAV infection. Treatment with inhibitors, for example, 3-MA[91], Bafilomycin A1^109^ and Chloroquine[110] suppressed IAV replication through inhibiting class III PI3K activity to block nucleation step of autophagy, inducing the accumulation of autophagosome and blocking the fusion of autophagosome and lysosome, respectively. Thus, natural compounds that regulate autophagy will have a great potential in anti-influenza virus therapy (Table 2). The research has shown that Astragaloside IV (AS-IV) originating from the astragalus root, weakened IAV-triggered the accumulation of autophagosomes and promoted autophagic flux for degrading viral particles[111]. In addition, Astragaloside IV sequestered pro- IL-1β into autophagosomes for degradation and blocking the secretion of IL-1β [111,112]. *Aloe vera* ethanol extract (AVE) inhibited IAV mRNA synthesis and protein expression as well as IAV-induced autophagy[113].

**Table 2.** Antiviral compounds related with autophagy against IAV

Antiviral compounds	Activity (IC50)	Virus subtype	Cell types	Mechanism of action	Literature
Astragaloside IV	50 mg/L	H1N1	A549	Promoting the fusion of autophagosomes with lysosome	111
Aloe vera ethanol extract (AVE)	25 μg/mL, 250 μg/mL	H1N1, H3N2	MDCK	Inhibiting IAV mRNA synthesis and protein expression	113
Catechin	50 μM	H1N1	A549	Decreasing the expression of viral M2 and NP protein	114
Tauroursodeoxycholic acid	3 mmol/L	H1N1, H5N1	A549	Interfering the oligomeric states of influenza viral M2	115
Salinomycin	10 μM	H1N1	MDCK	Inhibiting the proton channel activity of viral M2	116
Thiopurines	10 μM	H1N1	A549	Affecting the synthesis and maturation of IAV glycoproteins hemagglutinin and neuraminidase	117
Baicalin	50 μg/mL	H3N2	A549, Ana-1	Inhibiting the activity of IAV neuraminidase; inhibiting the activity of IAV neuraminidase	121,122,123,124
Punicalagin	10 μM	H1N1, H3N2	MDCK	An inhibitor of influenza neuraminidase and regulating Akt/FOXO3a and P62/Nrf2 signalling pathway to control autophagy activation	118,119
Atorvastatin	5 μM	H1N1	MDCK	Inhibiting HMG-CoA reductase activated by autophagy to block the replication of IAV	125
Anthocyanins	10 µg/mL	H1N1	MDCK	Suppressing influenza virus adsorption	126,127
Berberine	16.8 µM	H1N1	murine macrophage cell	Stimulating BNIP3-mediated mitophagy initiation to inhibit IAV-induced excessive inflammasome	133
Oligonol	10 µg/mL	H1N1	A549	Activating SIRT1-AMPK-autophagy pathway to block IAV replication	134, 135
Vitamin D3	100 nM	H1N1	A549	Increasing the expression of Syntaxin-17 (STX17), which may be an inhibitor of membrane fusion and mediate mitophagosome-lysosome fusion	126,137,138,139
Procyanidin	25 µg/mL	H1N1	A549	Leading to the accumulation of LC3II and inhibited the expression of autophagy-related proteins for blocking IAV replication	140
Evodiamine	12.5 µg/mL	H1N1	A549	Suppressing the dissociation of Beclin1-Bcl2 heterodimer	141
Eugenol	5 µg/mL	H1N1	A549	Inhibiting the Dissociation of Beclin1-Bcl2 Heterodimer and the Elevation of Autophagy Induced by IAV	142
Silybin	100 μM	H1N1	A549	Blocking the Atg12-Atg5/Atg16 heterotrimer, autophagosomes accumulation and oxidative stress	142
Edible bird’s nest	5 μg/ml	H1N1	MDCK	Decreasing the RhoA protein expression, reducing LC3-II protein level, and increasing the lysosomal degradation.	131
Hochuekkito	12.5 mg/ml	H1N1	MDCK	Preventing IAV-induced cell death via the induction of autophagy in host cell at the early post-infection stage	132

Some antiviral compounds directly targeted influenza viral proteins and then blocked the novel viral synthesis via autophagy machinery. Treatment of catechin led to the decrease of M2 and NP protein expression during IAV infection. Moreover, catechin could decrease autophagosome number to restore the formation of autolysosomes for degradation of viral particles, thereby inhibiting viral replication[114]. Tauroursodeoxycholic acid (TUDCA), an ER stress inhibitor, interfered the oligomeric states of influenza viral M2 resulting in its proton conductivity inactivity[115]. Salinomycin was also reported a key function against proton channel activity of viral M2, which induced the accumulation of autophagosome[116]. Thiopurines affected the synthesis and maturation of IAV glycoproteins hemagglutinin (HA) and neuraminidase (NA) via antiviral unfolded protein response[117]. Punicalagin, an inhibitor of influenza neuraminidase, controlled autophagy activation to inhibit IAV infection through regulating Akt/FOXO3a and p62/Nrf2 signalling pathway [118,119]. Taken together, these drugs inhibited the expression of viral protein to ameliorate viral protein-mediated autophagosome accumulation and transported the harmful substance into lysosome for degradation.

In addition, antiviral compounds utilized autophagy machinery to inhibit autophagy and then regulated the activity of host factors during IAV infection. An anti-enterovirus 71 (EV71) natural flavonoid, Chrysin, showed its anti-influenza virus activity via activating mTOR and inhibiting autophagy[120]. Baicalin, a natural product extracted from Scutellariaradix, has been reported to block IAV replication via inhibiting the activity of IAV neuraminidase and activating cellular innate immune responses [121–123]. Baicalin treatment inhibited the expression of LC3-II and Atg5-Atg12 complex that initiated autophagy formation, thereby suppressing IAV-induced autophagic degradation of innate immune factors, including MAVS[124]. Atorvastatin inhibited autophagy-induced HMG-CoA reductase during IAV infection, and then blocked the formation of lipid droplets and the replication of virus[125]. Anthocyanins, a natural antioxidant, showed the antiviral activity against IAV[126] and inhibited autophagic cell death[127]. Moreover, its major component cyanidin-3-glucoside (C3G) not only suppressed the expression of LC3II, Beclin1 and USP19, but also reduced autophagosome number and oxidative stress[128]. Down-regulation of USP19 increased the phosphorylation levels of TBK1 and IRF3 and inhibited autophagy induced by IAV infection due to the ubiquitination of Beclin1[129], indicating that Cyanidin and C3G showed anti-influenza virus activity against IAV infection by regulating USP19-Beclin1-dependent autophagy. Moreover, the compound cocktail and traditional medicine has been reported to have antiviral activities against IAV. The compound cocktail (Arctiin, Daidzein, Glycyrrhizic acid and Liquiritin) induced autophagic flux in natural killer cells[130]. A traditional Chinese medicine, Edible bird’s nest reduced LC3-II expression and induced the degradation of autophagy protein in lysosome in presence of IAV, suggesting that Edible bird’s nest utilized autophagy mechanisms to show its antiviral activity[131]. In addition, traditional Japanese herbal medicine, Hochuekkito promoted autolysosomes formation in IAV-infected cells at the early post-infection stage[132]. Although autophagy was involved in the antiviral activities of theses medicines against IAV infection, the mechanism of autophagy regulated by these medicines was not shown and needs to be clarified.

IAV infection usually induced higher concentrations of inflammatory cytokines and excessive inflammation, leading to host cell death. Autophagy dysfunction acted as the regulator of inflammasomes and caused severe disease. Berberine restricted NLRP3 inflammasome activation induced by IAV via increasing mitochondrial membrane potential and decreasing the generation of mitochondrial ROS, thus alleviating IAV-induced lung injury. Berberine stimulated BNIP3-mediated mitophagy initiation to inhibit IAV-induced excessive inflammasome[133]. Oligonol treatment attenuated ROS formation and pro-inflammatory IL-8 production to inhibit influenza infection by upregulating the expression SITR1 that has been shown an antiviral effect [134,135]. Additionally, Oligonol treatment not only increased the expression levels of LC3-II and the phosphorylation of AMPK, but also reduced p62/SQSTM1 levels in IAV-infected cells, suggesting that Oligonol activated SIRT1-AMPK-autophagy pathway to block IAV replication[135]. Vitamin D3 has been determined to mitigate IAV-induced inflammatory and its supplementation triggered the fusion of autophagosome-lysosome, thereby inhibiting IAV-induced apoptosis via increasing STX17 expression, which acted as an inhibitor of membrane fusion and mediates mitophagosome-lysosome fusion [42,136–138].

With the technologic development, High-throughput screens was used to identify the novel compounds against IAV. High-throughput screening (HTS) platform based on the influenza A virus (IAV) vRNA promoter has been constructed to identify multiple of traditional Chinese medicine, and future analysis showed that both procyanidin and evodiamine led to the accumulation of LC3II and inhibited the expression of autophagy-related proteins for blocking IAV replication [139,140]. In addition, eugenol from Syzygium aromaticum was identified to exhibit the best activity in inhibiting autophagy and viral replication based on bimolecular fluorescence complementation (BiFC) technique by analyzing the dissociation of Beclin1-Bcl2 heterodimer[141]. At the same time, based on the inhibition of Atg12-Atg5/Atg16 heterotrimer, silybin and its derivatives showed strongly anti-IAV activity via blocking the Atg12-Atg5/Atg16 heterotrimer, autophagosomes accumulation and oxidative stress[142]. Moreover, host factors required in IAV infection were identified by different screening strategies, for example, RNAi, CRISPR/Cas9. Therefore, these dependency factors involved in autophagy might also be drug targets to develop novel drugs against IAV infection. Taken together, abundant of drug compounds can be used as autophagy-related anti-influenza virus drug against IAV replication or IAV-induced inflammatory.

## Conclusion

Autophagy was the key to two hostile forces, host and virus for survival. The initial role of autophagy was targeting the damaged organelles, protein aggregates and intracellular infectious pathogens for lysosomal degradation to maintain cellular homeostasis, but virus has evolved specific strategies to regulate the autophagic process for viral infection. Following IAV infection, host resistance factors were activated, or host factors required in IAV infection were inactivated to initiate autophagy to curtail infection via disturbing viral proteins synthesis, delivering viral particles for lysosomal degradation and inhibiting IAV-induced inflammatory. Conversely, in order to successfully facilitate viral infection and pathogenesis, IAV not only induced complete autophagy early in the life cycle to affect host antiviral immune, but also blocked autophagic flux at a late stage of infection to block the degradation of viral particle by utilizing the specific roles of difference viral proteins. Moreover, IAV infection could also hijack autophagy to degrade or inactivate host immunity-related proteins for evading host immunity responses. Currently, multiple of drugs based on autophagic antiviral activity were exploited in response to IAV infection and our review summarized the molecular mechanism of drugs against IAV infection, including the vaccination protocols with nanoparticles.

Although some mechanisms of host and IAV-regulated autophagy had been uncovered well decades earlier, understanding about the battle between host and influenza virus is still quite rough, for example, how host inhibit host pro-viral factors required for IAV replication via autophagy-dependent degradation or activate host resistance factors to regulate autophagy for blocking IAV infection, how the coordination between influenza viral proteins regulates autophagy to support its spread.
